# Development of Genotype-Specific Anti-Bovine Rotavirus A Immunoglobulin Yolk Based on a Current Molecular Epidemiological Analysis of Bovine Rotaviruses A Collected in Japan during 2017–2020

**DOI:** 10.3390/v12121386

**Published:** 2020-12-03

**Authors:** Koki Odagiri, Nobuki Yoshizawa, Hisae Sakihara, Koji Umeda, Shofiqur Rahman, Sa Van Nguyen, Tohru Suzuki

**Affiliations:** 1 Immunology Research Institute in Gifu, EW Nutrition Japan K.K., Gifu 501-1101, Japan; odagiri@ew-nutrition.co.jp (K.O.); sakihara@ew-nutrition.co.jp (H.S.); umeda@ew-nutrition.co.jp (K.U.); rahman@ew-nutrition.co.jp (S.R.); nguyen@ew-nutrition.co.jp (S.V.N.); 2 Division of Pathology and Pathophysiology, Hokkaido Research Station, National Institute of Animal Health, NARO, Sapporo, Hokkaido 062-0045, Japan; yoshizawan334@affrc.go.jp; 3 Ehime Prefectural Livestock Disease Diagnostic Center, Toon, Ehime 791-0212, Japan

**Keywords:** calf, diarrhea, bovine rotavirus A, VP4 and VP7, G and P genotypes, immunoglobulin yolk, passive immunization

## Abstract

Bovine rotavirus A (RVA), a major causative pathogen of diarrhea in dairy and Japanese beef calves, has led to severe economic losses in numerous countries. A dual genotyping system based on genomic segments encoding VP7 (G genotype) and VP4 (P genotype), comprising the outer layer of the virion, has been used to understand the epidemiological dynamics of RVAs at the national and global levels. This study aimed to investigate occurrence frequency of G and P genotypes for multiple bovine RVAs from calf diarrheic samples collected in Japan from 2017 to 2020. After we produced anti-bovine RVA immunoglobulin yolks (IgYs) from hens immunized with the two RVAs with different genotypes (G6P[5] and G10P[11]) selected on the basis of the current epidemiological survey, we investigated cross-reactivity against bovine RVAs with different G and P combinations owing to establish a useful strategy to protect calves from RVA infections using the two IgYs. Consequently, the two produced anti-bovine IgYs showed strong cross-reactivity against bovine RVAs with the same G and/or P genotypes in neutralization assay, respectively. Therefore, our data suggest the possibility of a passive immunization to protect calves from a bovine RVA infections epidemic in Japan via oral administration of the two IgYs into calves. The findings presented herein will provide important information that IgY is one of the effective tools to prevent infections of various pathogens.

## 1. Introduction

Young calves are easily prone to pathogen infections owing to their developing immune system and immature immune response. In Japan, the economic losses due to diarrhea in calves are estimated at approximately one billion yen per year according to the 2017 annual report from the Ministry of Agriculture, Forestry and Fisheries of Japan [1]. The major causative agents of diarrhea in young calves are commonly considered to be bovine rotavirus A (RVA), *Escherichia coli*, *Cryptosporidium* spp., and *Eimeria* spp. [2,3,4]. Furthermore, our previous study demonstrated that bovine RVAs have been most frequently (approximately 20% each) detected in diarrhea samples from dairy and Japanese beef calves [5]. Therefore, the total amount of economic losses caused by RVA infections in calves is estimated at approximately two hundred million yen per year in Japan, which would be an enormous problem.

Rotaviruses, members of the family *Reoviridae*, are a major causative pathogen of diarrhea in humans and animals, involving the deaths of 200,000 children in developing countries and causing economic losses in the livestock industry globally [6,7]. They are currently divided into twelve species (RVA to rotavirus L (RVL)) on the basis of the sequence diversity of the inner capsid protein VP6 [8,9,10,11]. The pathogen has 11 double-stranded RNA (dsRNA) segments encoding six viral proteins (VP1–4, VP6, and VP7) and five or six nonstructural proteins (NSP1–6). VPs consist of infectious triple-layered particles surrounding the genomic dsRNA. NSPs are primarily associated with dsRNA replication and transcription, cellular pathogenesis, and virus-particle maturation [12].

The two outer capsid proteins, VP7 and VP4, induce neutralizing antibodies and form the basis for G and P genotype assignment [6]. A dual genotyping system based on the genomic segments encoding VP7 and VP4 has been used to understand the epidemiological dynamics of RVAs at the national and global levels. The G and P genotypes are commonly dependent on host species, because rotavirus has host-specific barriers and restrictions. However, human rotaviruses with unusual G and P genotypes have emerged owing to interspecies transmissions and natural reassortments between humans and animals, especially cows and pigs [13,14,15,16,17]. Therefore, it is important to continuously monitor emergence of a new rotavirus in animals to prevent their transmission between humans and animals.

Among bovine RVAs, three G genotypes (G6, G8, and G10) and three P genotypes (P[1], P[5], and P[11]) are the most common [6]. Besides them, so far, bovine RVAs with additional unusual G genotypes (G15, G18, G21, and G24) and P genotypes (P[14], P[17], P[29], and P[33]) have been detected in Japan. In particular, G18 and P[17] genotypes were originally identified in an avian RVA; however, the avian-like RVA has also been detected from calves in Germany and Japan [18,19]. Abe et al. reported the isolation of bovine RVAs with G21P[29] and G24P[33] genotypes from asymptomatic cows in Japan [20,21]. To our knowledge, however, little epidemiological studies have been recently performed on bovine RVAs in Japan.

Passive immunization by using oral administration of immunoglobulin yolk (IgY) originated from hyperimmune chicken has been reported to be effective against infectious diseases by virus, bacteria, fungi, and protozoa in both humans and animals [22,23,24]. Especially, there have been reports on the experimental usage of rotavirus specific IgYs in cats, mice, and cows [25,26,27,28]. Therefore, IgY could be fully expected as one of the useful strategies to protect calves from RVA infections.

In the present study, we attempted to survey the current epidemiological dynamics of bovine RVAs in Japan via sequence and phylogenetic analyses using diarrheic samples from dairy and Japanese beef calves collected in Japan during 2017–2020. Based on our survey, we attempted to produce anti-bovine RVA IgYs against two dominant genotypes of bovine RVAs circulating in Japan and validate cross-reactivity for bovine RVAs with different genotypes via neutralization assay using the two IgYs. Consequently, we succeeded to produce IgYs with enough cross-reactivity for epidemic bovine RVAs circulating in Japan. Passive immunization into cattle orally administered those IgYs will be useful for reduction of economic losses owing to diarrhea in the cattle industry.

## 2. Materials and Methods

### 2.1. Samples, RNA Extraction, and RT-PCR Amplification

Ninety-nine diarrheic samples from calves aged between 5 and 91 days were collected at 62 farms in 8 prefectures in Japan during February 2017 to May 2020 (Table 1). The viral RNA was extracted from 10% fecal suspension in minimum essential medium using TRIzol LS (Thermo Fisher Scientific, Carlsbad, CA, USA), according to the manufacturer’s instructions. The nearly full-length nucleotide sequences of the VP7 and VP4 segments from the 99 bovine RVAs were amplified by reverse-transcription-polymerase chain reaction (RT-PCR) using primers that modified the RVA universal primers reported by Fujii et al. [29] as follows: VP7-F, GGCTTTAAAAGMGAGAATTTCCGWYTGGC (nucleotide (nt) position: 1–29); VP7-R, GGGTCACATCATACARYTCTAAYYAA (nt position: 1038–1062); VP4-F, GGGCTATAAAATGGCTTCKCTCATWTA (nt position: 1–27); and VP4-R, GGTCACATCCTCYAGMMACTRC (nt position: 2341–2362). RT-PCR was carried out using a PrimeScript II High Fidelity One Step RT-PCR kit ver.2 (Takara, Shiga, Japan), according to the following conditions: 45 °C for 10 min and 70 °C for 15 min; 35 cycles of 98 °C for 10 s, 55 °C for 15 s, and 68 °C 2 min; and a final extension at 68 °C for 7 min.

### 2.2. Sequence and Phylogenetic Analyses

PCR products were sequenced using a BigDye Terminator v3.1 Cycles Sequencing Kit on an automated ABI Prism 3130 Genetic Analyzer (Thermo Fisher Scientific, Carlsbad, CA, USA). Each genomic sequence from the 99 bovine RVA strains determined herein was submitted to the DNA Data Bank of Japan (DDBJ); the sequences are retrievable from GenBank (LC590915–LC591102, Table 1). The sequence data were aligned using the ClustalW method in the MEGA X program [30]. Phylogenetic analyses were conducted using the maximum-likelihood method with the general time reversible nucleotide substitution model and 1000 bootstrap replicates. Genotype classifications of the VP7 and VP4 genes from the 99 bovine RVA strains were conducted using 80% cutoff values calculated in a previous study [31].

### 2.3. Virus Isolation

MA-104 cells (Rhesus monkey fetal kidney cells: ATCC, CRL-2378) were maintained by using Eagle’s Minimal Essential Medium (EMEM) (Nissui, Tokyo, Japan) supplemented with 10% fetal bovine serum (FBS) in our laboratory.

Representative bovine RVAs were isolated from each fecal sample according to the methods as previously described, with modifications [32]. Briefly, fecal samples were homogenized with serum-free EMEM and centrifuged at 2100× *g* for 15 min at 4 °C to remove debris. The supernatant was filtered through a 0.45 µm membrane (Millipore, Darmstadt, Germany) and treated with 10 µg/mL trypsin from bovine pancreas (Sigma Chemicals, MO, USA) at 37 °C for 1 h. Then, 200 µL of the treated supernatant were inoculated into monolayers of the MA-104 cells (2.2 × 10^5^ cells/mL) in glass tube (4 tubes per each isolate) and kept at 37 °C for 90 min. Thereafter, inoculum was removed from the cells, fed with EMEM containing 1.5 µg/mL trypsin, and incubated at 37 °C for 3 days. When cytopathic effects (CPEs) were observed by microscopy, the supernatant was harvested and repeatedly inoculated into newly prepared MA-104 cells until forth passage. The virus titers (a 50% tissue culture infective dose (TCID_50_)/mL) were determined according to the method reported by Reed and Muench with fourth replicates [33].

### 2.4. Production of Anti-Bovine Rotavirus A Immunoglobulin Y and Control Immunoglobulin Y

All procedures involving animals were approved by the animal care and use committee of EW Nutrition Japan K.K. (EWNJ protocol number 20190401). We chose two representative bovine RVA strains, OKY31 (G10P[11]) and SMN35 (G6P[5]), based on our current survey described above, as antigens for the production of anti-bovine RVA IgY, according to the methods described previously [34]. Prior to their use as immunizing antigen, the two bovine RVA strains were inactivated using 0.3% formalin at 37 °C for 24 h. Five 5-month-old White Leghorn hens (HyLine W36 strain produced by GHEN Corporation, Gifu, Japan) kept in conventional isolated poultry housing were immunized with the two inactivated RVAs, respectively. The hens were injected intramuscularly in the breast muscles with 1.0 mL of mixture (0.5 mL in each breast muscle) of inactivated virus suspension of 10^9.0^ TCID_50_/mL with an equal volume of Freund’s Incomplete Adjuvant (FICA) (Becton Dickinson, MD, USA). Eggs laid by the immunized hens between 3 and 10 weeks after immunization were harvested and egg yolk was isolated, pooled, and processed into powder form in accordance with a method described previously [35]. Control IgY powder was prepared according to the same method from the eggs of hens immunized with culture medium from mock-infected MA-104 cell monolayer. For neutralization assay, two anti-bovine RVA IgYs and control IgY were partially purified from egg yolk by chloroform extraction and ammonium sulfate precipitation [36]. The antigen and antibody protein concentrations were determined with Bio-Rad protein assay (Bio-Rad Laboratories, Hercules, CA, USA).

### 2.5. Neutralization Assay

Ten bovine RVA strains with different G and P genotypes (1 bovine RVA strain, SMN-1 with G6P[1], 2 bovine RVA strains, HKD18 and SMN35 with G6P[5], 3 bovine RVA strains, HKD6, HKD7, and HKD17 with G6P[11], 2 bovine RVA strains, KK-3 and OKY31 with G10P[11], 1 bovine RVA strain, MYG-1 with G8P[14], and 1 bovine RVA strain, Dai-10 with G24P[33]) have been used in neutralization assay (Table S1). Six bovine RVA strains (HKD6, HKD7, HKD17, HKD18, OKY31, and SMN35) were isolated in the present study. Two bovine RVA strains (SMN-1 and KK-3), originally provided from the National Institute of Animal Health (Tsukuba, Ibaraki, Japan), were maintained in our laboratory [37]. The two remaining bovine RVA strains (MYG-1 and Dai-10) were kindly gifted from Dr. Matsuo, and Dr. Sugiyama from the Sendai Livestock Hygiene Center (Sendai, Miyagi, Japan), and Gifu University (Gifu, Gifu, Japan), respectively.

MA-104 cells (2.2 × 10^5^ cells/mL) were incubated at 37 °C for 72 h to prepare a confluent monolayer in glass tubes. IgY samples were heat-inactivated at 56 °C for 30 min and then diluted serially twofold from 1:10 with serum-free EMEM containing 1.5 µg/mL trypsin. The diluted IgYs were mixed with an equal volume (500 µL) of virus solution containing 200 TCID_50_ of each bovine RVA strain, respectively. The mixture was incubated at 37 °C for 1 h. After incubation, 100 µL of the mixture at each dilution were added in quadruplicate to a tube (4 tubes per dilution) containing a confluent MA-104 cell monolayer. IgY-virus mixture was allowed to adsorb at 37 °C for 90 min. The culture was fed with serum-free EMEM containing 1.5 µg/mL trypsin and then incubated at 37 °C for 7 days. The end-point titers were calculated according to the Reed and Muench method, with four replicates for titration [33]. The highest IgY dilution that protected more than the 50% of cells from CPE was taken as the neutralization antibody titer, which was expressed as the reciprocal of the highest IgY dilution that showed inhibition of CPE. The neutralization antibody titer was calculated from 5 independent assays.

### 2.6. Statistical Analysis

The frequency of occurrence of G and P genotypes of bovine RVAs in each cattle type was statistically analyzed using Fisher’s exact test with the R software (version 4.0.3). *P* value of under 0.05 indicated a significant difference.

## 3. Results

### 3.1. Sequence and Phylogenetic Analysis of VP7

We succeeded in determining the VP7 open reading frame (ORF) nucleotide sequences from 97 of 99 bovine RVA strains with a pair of modified universal RVA primers (Table 1). Comparative sequence analysis among the VP7 ORFs from the 97 bovine RVAs showed that the VP7 ORFs all had the same length (981 nucleotides) without deletions or insertions, except for 7 bovine RVAs with partial ORF sequences.

**Figure 1:**
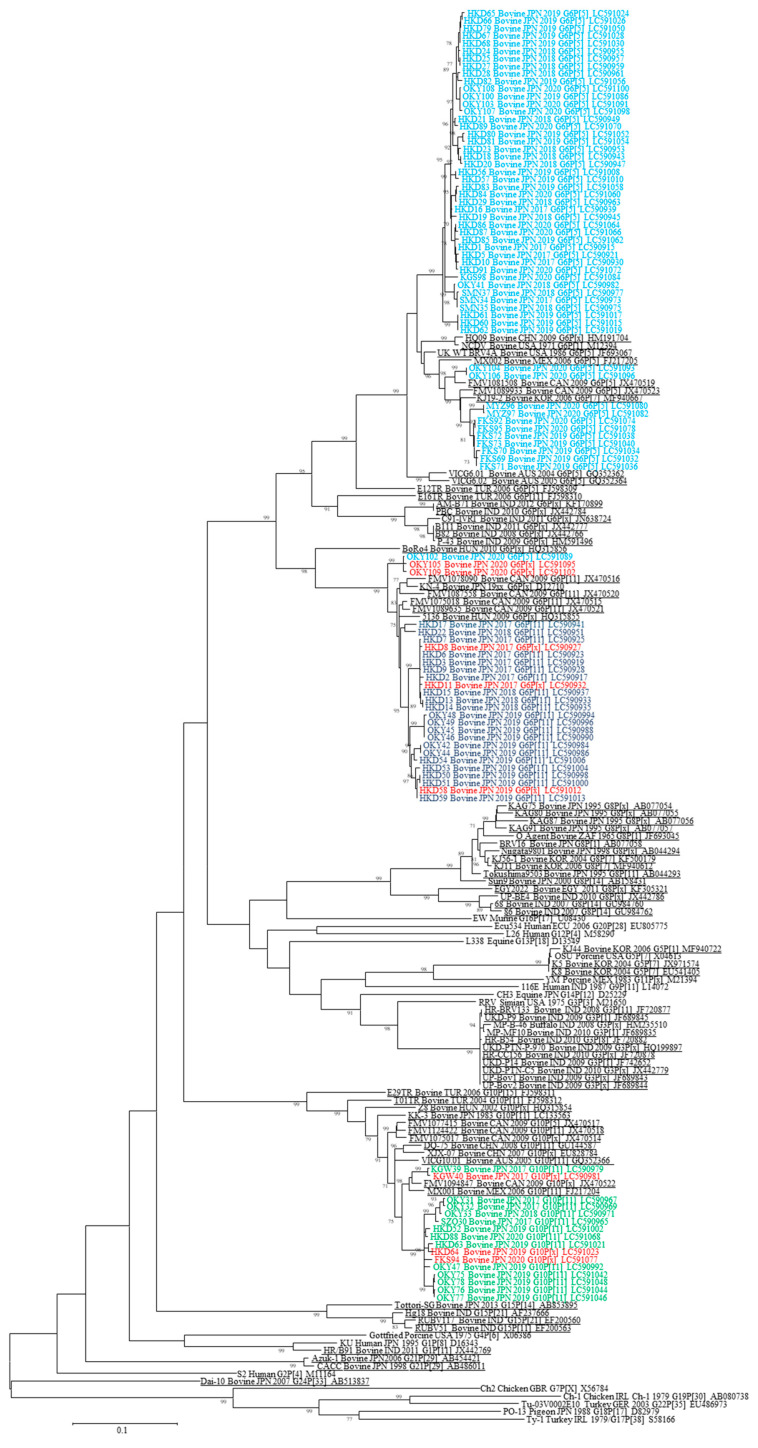
Phylogenetic tree based on the open reading frame (ORF) nucleotide sequences of rotavirus A (RVA) VP7. A phylogenetic tree was constructed using the maximum-likelihood method with MEGA X software. The number beside each node represents the percentage bootstrap support of 1000 replicates for the cluster. Bootstrap values <70% are not shown. Genotype classification was performed using a cutoff value of 80% defined by the Rotavirus Classification Working Group. The strains shown in colors and underlined indicate the bovine RVAs analyzed in this study and those reported in earlier studies, respectively. Different colors represent RVAs with different combinations of G and P genotypes. Host species, source countries, collection years, G and P genotypes, and GenBank accession numbers are shown following the strains.

Phylogenetic analysis of the VP7 segment was performed with the cutoff value of 80% using all RVA strains, i.e., combining the 97 bovine RVA strains with other bovine RVA strains available in GenBank, and the representative RVA strains belonging to each genotype (G1–G22) (Figure 1). Our analysis revealed that the 99 bovine RVA strains were classified into G6 (81/99: 82%), G10 (16/99: 16%), and G[x] (2/99: 2%) genotypes.

### 3.2. Sequence and Phylogenetic Analysis of VP4

Nearly full-length VP4 ORF nucleotide sequences except nucleotides at the 5′ and 3′ terminals from 91 of 99 bovine RVA strains were determined by using a set of originally designed primers (Table 1). There were no deletions or insertions compared to VP4 ORF nucleotide sequences from the 91 bovine RVAs.

**Figure 2:**
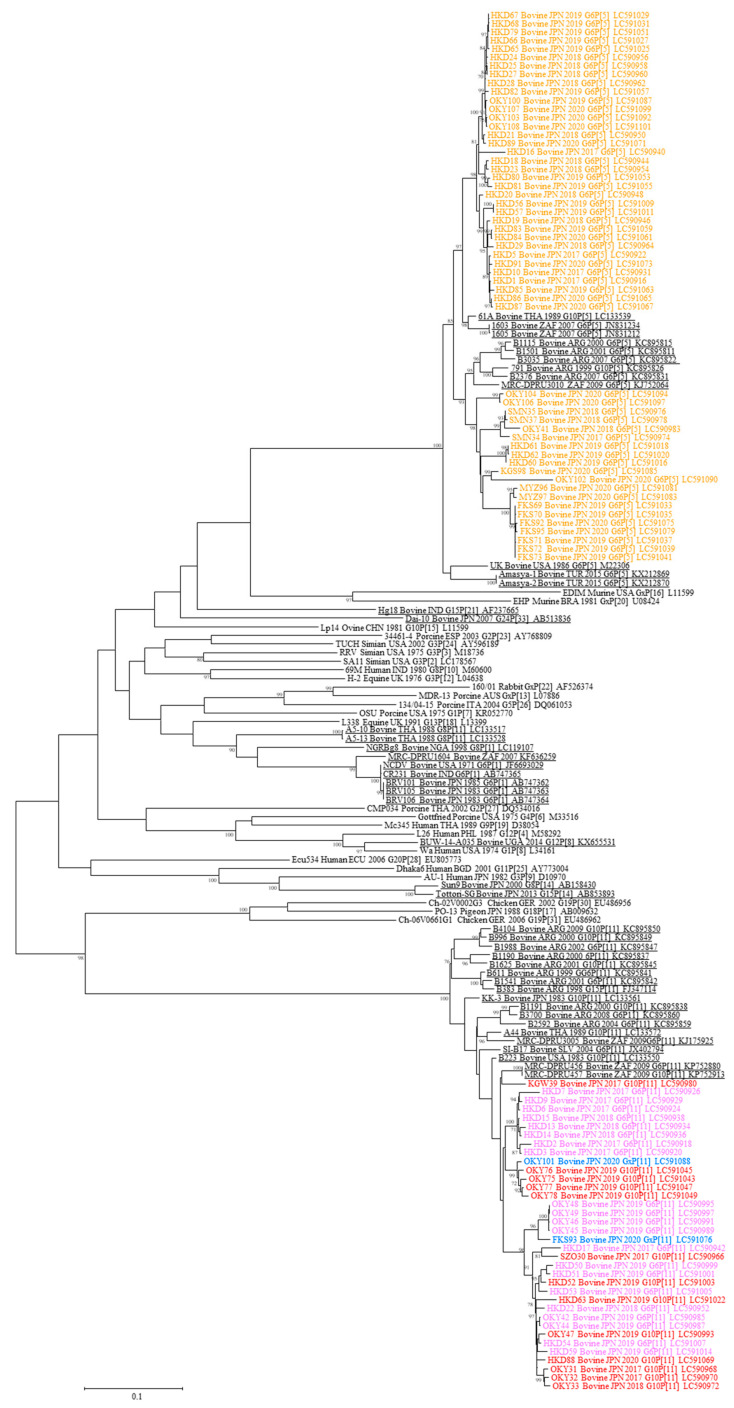
Phylogenetic tree based on the open reading frame (ORF) nucleotide sequences of rotavirus A (RVA) VP4. A phylogenetic tree was constructed using the maximum-likelihood method with MEGA X software. The number beside each node represents the percentage bootstrap support of 1000 replicates for the cluster. Bootstrap values <70% are not shown. Genotype classification was performed using a cutoff value of 80% defined by the Rotavirus Classification Working Group. The strains shown in colors and underlined indicate the bovine RVAs analyzed in this study and those reported in earlier studies, respectively. Different colors represent RVAs with different combinations of G and P genotypes. Host species, source countries, collection years, G and P genotypes, and GenBank accession numbers are shown following the strains.

Genetic classification of the VP4 segment was carried out using the data that added the VP4 ORF nucleotide sequences from the 91 bovine RVA strains to those of other bovine RVA strains and the representative RVA strains from each P genotype (P[1]–P[31]) according to the cutoff value of 80% at the nucleotide level (Figure 2). As a result, the 99 bovine RVA strains were differentiated into P[5] (55/99: 56%), P[11] (36/99: 36%), and P[x] (8/99: 8%).

### 3.3. Analysis of the Combination of G and P Genotypes

**Figure 3:**
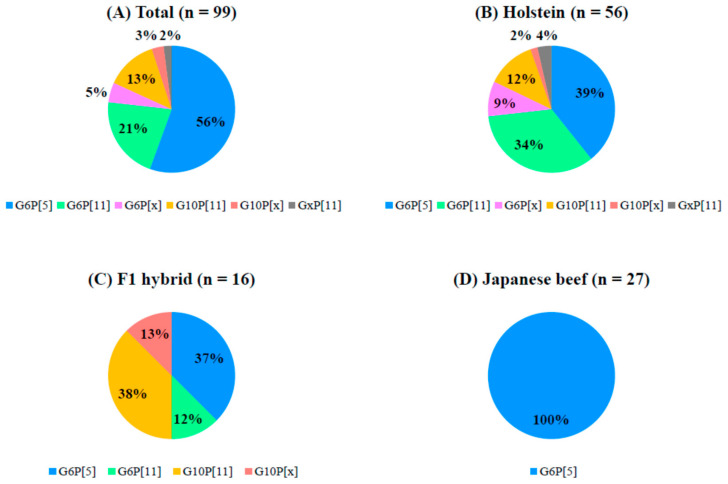
Frequency (in percent) of the occurrence of G and P genotypes of bovine rotaviruses A in each cattle type. (**A**) Total, (**B**) Holstein calves, (**C**) F1 hybrids, and (**D**) Japanese beef calves. Different combinations of G and P genotypes are shown in different colors.

As a whole, the frequencies of occurrence of G and P genotypes among 99 bovine RVA strains used in this study were 55 for G6P[5] (56%), 21 for G6P[11] (21%), 13 for G10P[11] (13%), 5 for G6P[x] (5%), 3 for G10P[x] (3%), and 2 for GxP[11] (2%) (Figure 3). Furthermore, our analysis revealed that there were clear differences (*p* < 0.05) in the frequency of occurrence of G and P genotypes according to cattle type. Briefly, G6P[5] and G6P[11], and G6P[5] and G10P[11] were the most predominant in Holsteins and F1 hybrids. In addition, all of the bovine RVAs (100%) detected in Japanese beef cattle had the G6P[5] genotype.

### 3.4. Production of Anti-Bovine Rotavirus A Immunoglobulin Y and Control Immunoglobulin Y

Anti-SMN35 and OKY31 IgYs, and control IgY were partially purified from egg yolks from chickens immunized with two bovine RVAs, and culture medium from mock-infected MA-104 cells, respectively, and measured their protein concentrations using Bio-Rad protein assay. Protein concentrations of anti-SMN35 and OKY31 IgYs, and control IgY were recorded as 40.1, 53.4, and 30.3 mg/mL, respectively. Thereafter, the measured protein concentration of each IgY sample was adjusted with 10 mg/mL for the neutralization assay.

### 3.5. Neutralization Assay

Two anti-bovine RVA IgYs and one control IgY were investigated for cross-reactivity against the ten isolated bovine RVA strains*,* homologous and heterologous with the two via neutralization assay (Table S1 and Table 2). Anti-SMN35 IgY showed strong cross-reactivity against six bovine RVA strains with G6 genotype, but lower cross-reactivity of 8- to >64-fold than those of the homologous neutralization antibody titers against the four remaining bovine RVA strains with different G genotypes. In contrast, anti-OKY31 IgY exhibited strong cross-reactivity against two bovine RVA strains with the same G and P genotypes (G10P[11]), and cross-reactivity of 4-fold reduction of the homologous neutralization antibody titers against three bovine RVA strains with the same P genotype (P[11]). Moreover, anti-OKY31 IgY had cross-reactivity of 4- to >32-fold lower than those of the homologous neutralization antibody titers against the five remaining bovine RVA strains with different G and P genotypes. Control IgY showed no cross-reaction (<20) with all bovine RVA strains.

## 4. Discussion

To investigate the current epidemiological dynamics of bovine RVAs in Japan, we analyzed diarrheic samples from 99 calves collected from multiple farms during 2017–2020 by using the dual genotyping system based on the genomic segments encoding VP7 and VP4. In the genotyping of VP7 and VP4, 99 bovine RVAs were classified into G6 and G10 within the G genotype, and P[5] and P[11] within the P genotypes of the three most common genotypes, respectively [15]. Other G genotypes (G8, G15, G18, G21, and G24) and P genotypes (P[1], P[14], P[17], P[29], and P[33]) distributed in Japan were not identified in this study [18,20,21,38,39,40]. Although bovine RVAs with G8 genotypes were frequently identified from calf diarrheic samples in Japan in the 1990s, they were hardly detected in a surveillance of bovine RVAs in diarrheic samples from calves collected from 1987 to 2000 [39,40,41,42,43]. Therefore, these data suggest that bovine RVAs with G8 genotypes were transiently prevalent in Japan in the 1990s. In fact, our data indicate that they have not been predominantly distributed in Japan in recent years. Although bovine RVAs with G21P[29] and G24P[33] genotypes have been isolated from asymptomatic cows in Japan during 2006–2008, our analysis could not detect those unique bovine RVAs [20,21]. This might be ascribed to the differences in samples from asymptomatic and diarrheic calves. However, unique bovine RVAs might still be maintained and transmitted among healthy cattle herds. This possibility needs to be verified using more samples, particularly fecal samples from healthy cattle in future studies.

In the analysis for the combination of G and P genotypes, bovine RVAs with G6P[5] (56%), G6P[11] (21%), and G10P[11] (13%) have been widely distributed throughout Japan during 2017–2020. In addition, bovine RVAs with G6P[5] have been mainly identified in Japanese beef cattle. Moreover, bovine RVAs with G6P[11] and G10P[11], in addition to G6P[5], respectively, have been dominantly detected in Holstein and F1 hybrid cattle. The relationship between bovine RVA and cattle type might be closely associated with the breeding, feeding, and movement of cows, which depend on cattle type, and/or host-virus interaction. To clarify this possibility, further continuous surveillance is warranted on the spread of bovine RVAs in Japan.

Several reports on the molecular epidemiology of bovine RVAs in calves distributed in Japan have been published so far [41,43]. A continuous surveillance of bovine RVAs using diarrheic samples from calves collected in Kagoshima prefecture during 1995–1998 showed that bovine RVAs with G10P[11], G8P[X], and G6P[5] were predominantly distributed in 1995, 1996, and both 1997 and 1998, respectively [41]. On the other hand, a systematic surveillance of bovine RVAs in diarrheic samples of calves collected from 29 dairy and Japanese beef farms in 11 prefectures from 1987 to 2000 indicated the existence of bovine RVAs with multiple genotypes of G6P[5] (37%), G10P[11] (30%), G6P[1] (11%), G6P[11] (11%), G10P[5] (9%), and G8P[11] (1%) [43]. Compared with previous studies, our data demonstrated that bovine RVAs with G6P[5] and G10P[11] genotypes have been maintained in cattle herds for over 30 years, and bovine RVAs with G6P[11] genotype have been recently widespread in cattle herds.

In the present study, we produced two different types of IgY based on our current surveillance for bovine RVAs distributed in Japan, and investigated cross-reactivity for bovine RVAs with several different G and P genotypes using the two IgYs. One antibody (anti-SMN35 IgY: G6P[5]) showed broad cross-reactivity against six bovine RVA strains with the same G6 genotype, but lower cross-reactivity (8- to >64-fold reduction) than those of the homologous neutralization antibody titers against bovine RVAs with different G genotypes. Another antibody (anti-OKY31 IgY: G10P[11]) highly cross-reacted with bovine RVAs with identical G and P genotypes, and did with bovine RVAs with the same P genotypes at the level of 4-fold reduction of the homologous neutralization antibody titers. In addition, our findings reveal that the two produced anti-bovine RVA IgYs have a potential to be a useful tool to control bovine RVAs in cattle herd dominantly circulating in Japan, because oral administration of antibody with neutralization antibody titers of over 320 can provide a sufficient passive immunization to calves as reported in previous studies [44,45]. Furthermore, the cross-reactivity profile presented herein suggests that a combination of multivalent anti-bovine RVA IgYs can provide a broad-spectrum passive immunization to calves and cows infected with them, considering the broad genotype diversity of bovine RVAs distributed in different regions of the world.

In conclusion, we presented the current epidemiological dynamics of bovine RVAs using multiple diarrheic samples of calves collected in recent years. Our data demonstrated that bovine RVAs with multiple genotypes have been maintained and transmitted among herds from various cattle types. Moreover, order-made production of IgY based on the epidemiological survey can be a new strategy to control and prevent epidemic bovine RVAs. These insights would be useful for prompting an application of IgY to protect calves from enteric viruses other than hygiene management.

## Figures and Tables

**Table 1:** Summary of individual information, and VP7 (G) and VP4 (P) genotypes of 99 bovine rotavirus A strains collected in Japan from 2017 to 2020.

Strain	Collection Area	Collection Data	Age	Cattle Type	VP7			VP4		
					Open Reading Frame (Length)	G Genotype ^a^	Accession Number	Open Reading Frame (Length)	P Genotype ^b^	Accession Number
HKD1	Hokkaido	2017.4	5	Holstein	981	G6	LC590915	2136	P[5]	LC590916
HKD2	Hokkaido	2017.4	10	Holstein	981	G6	LC590917	2226	P[11]	LC590918
HKD3	Hokkaido	2017.3	8	Holstein	981	G6	LC590919	2277	P[11]	LC590920
HKD5	Hokkaido	2017.3	7	Holstein	981	G6	LC590921	2283	P[5]	LC590922
HKD6	Hokkaido	2017.3	7	Holstein	981	G6	LC590923	2202	P[11]	LC590924
HKD7	Hokkaido	2017.3	9	Holstein	981	G6	LC590925	2199	P[11]	LC590926
HKD8	Hokkaido	2017.3	8	Holstein	981	G6	LC590927	N.D.	P[x]	-
HKD9	Hokkaido	2017.3	7	Holstein	981	G6	LC590928	2196	P[11]	LC590929
HKD10	Hokkaido	2017.5	4	Holstein	981	G6	LC590930	2286	P[5]	LC590931
HKD11	Hokkaido	2017.2	6	Holstein	981	G6	LC590932	N.D.	P[x]	-
HKD13	Hokkaido	2018.7	<7	Holstein	981	G6	LC590933	2274	P[11]	LC590934
HKD14	Hokkaido	2018.8	<7	Holstein	981	G6	LC590935	2274	P[11]	LC590936
HKD15	Hokkaido	2018.8	<7	Holstein	981	G6	LC590937	2274	P[11]	LC590938
HKD16	Hokkaido	2017.12	10	Holstein	981	G6	LC590939	2268	P[5]	LC590940
HKD17	Hokkaido	2017.12	12	Holstein	981	G6	LC590941	2235	P[11]	LC590942
HKD18	Hokkaido	2018.3	<7	Holstein	981	G6	LC590943	2274	P[5]	LC590944
HKD19	Hokkaido	2018.3	<7	Holstein	981	G6	LC590945	2277	P[5]	LC590946
HKD20	Hokkaido	2018.3	<7	Holstein	981	G6	LC590947	2277	P[5]	LC590948
HKD21	Hokkaido	2018.3	<7	Holstein	981	G6	LC590949	2283	P[5]	LC590950
HKD22	Hokkaido	2018.3	10	Holstein	981	G6	LC590951	2274	P[11]	LC590952
HKD23	Hokkaido	2018.3	8	Holstein	981	G6	LC590953	2280	P[5]	LC590954
HKD24	Hokkaido	2018.3	<7	Japanese beef	981	G6	LC590955	2265	P[5]	LC590956
HKD25	Hokkaido	2018.3	<7	Japanese beef	981	G6	LC590957	2271	P[5]	LC590958
HKD27	Hokkaido	2018.3	<7	Japanese beef	981	G6	LC590959	2274	P[5]	LC590960
HKD28	Hokkaido	2018.4	9	Holstein	981	G6	LC590961	2277	P[5]	LC590962
HKD29	Hokkaido	2018.4	9	Japanese beef	981	G6	LC590963	2277	P[5]	LC590964
SZO30	Shizuoka	2017.12	14	F1 hybrid	981	G10	LC590965	2265	P[11]	LC590966
OKY31	Okayama	2017.10	9	F1 hybrid	981	G10	LC590967	2268	P[11]	LC590968
OKY32	Okayama	2017.11	91	Holstein	981	G10	LC590969	2268	P[11]	LC590970
OKY33	Okayama	2018.1	62	F1 hybrid	981	G10	LC590971	2274	P[11]	LC590972
SMN34	Shimane	2017.11	10	F1 hybrid	981	G6	LC590973	2277	P[5]	LC590974
SMN35	Shimane	2018.1	13	Japanese beef	981	G6	LC590975	2286	P[5]	LC590976
SMN37	Shimane	2018.3	7	Japanese beef	981	G6	LC590977	2262	P[5]	LC590978
KGW39	Kagawa	2017.6	6	F1 hybrid	981	G10	LC590979	2268	P[11]	LC590980
KGW40	Kagawa	2017.7	9	F1 hybrid	981	G10	LC590981	N.D.	P[x]	-
OKY41	Okayama	2018.4	5	F1 hybrid	981	G6	LC590982	2271	P[5]	LC590983
OKY42	Okayama	2019.1	14	Holstein	981	G6	LC590984	2277	P[11]	LC590985
OKY44	Okayama	2019.2	15	F1 hybrid	981	G6	LC590986	2277	P[11]	LC590987
OKY45	Okayama	2019.1	10	Holstein	981	G6	LC590988	2277	P[11]	LC590989
OKY46	Okayama	2019.1	16	Holstein	981	G6	LC590990	2277	P[11]	LC590991
OKY47	Okayama	2019.1	20	F1 hybrid	981	G10	LC590992	2277	P[11]	LC590993
OKY48	Okayama	2019.2	39	F1 hybrid	981	G6	LC590994	2277	P[11]	LC590995
OKY49	Okayama	2019.2	13	Holstein	981	G6	LC590996	2277	P[11]	LC590997
HKD50	Hokkaido	2019.1	17	Holstein	981	G6	LC590998	2271	P[11]	LC590999
HKD51	Hokkaido	2019.1	15	Holstein	981	G6	LC591000	2271	P[11]	LC591001
HKD52	Hokkaido	2019.6	17	Holstein	975	G10	LC591002	2271	P[11]	LC591003
HKD53	Hokkaido	2019.2	9-11	Holstein	981	G6	LC591004	2271	P[11]	LC591005
HKD54	Hokkaido	2019.2	9-11	Holstein	981	G6	LC591006	2271	P[11]	LC591007
HKD56	Hokkaido	2019.3	8	Japanese beef	981	G6	LC591008	2289	P[5]	LC591009
HKD57	Hokkaido	2019.3	24	Japanese beef	969	G6	LC591010	2289	P[5]	LC591011
HKD58	Hokkaido	2019.3	14	Holstein	981	G6	LC591012	N.D.	P[x]	-
HKD59	Hokkaido	2019.3	10	Holstein	981	G6	LC591013	2271	P[11]	LC591014
HKD60	Hokkaido	2019.4	8	Holstein	981	G6	LC591015	2289	P[5]	LC591016
HKD61	Hokkaido	2019.4	8	Holstein	981	G6	LC591017	2289	P[5]	LC591018
HKD62	Hokkaido	2019.4	7	Holstein	981	G6	LC591019	2289	P[5]	LC591020
HKD63	Hokkaido	2019.5	<7	Holstein	981	G10	LC591021	2271	P[11]	LC591022
HKD64	Hokkaido	2019.5	<7	Holstein	981	G10	LC591023	N.D.	P[x]	-
HKD65	Hokkaido	2019.5	<7	Holstein	981	G6	LC591024	2289	P[5]	LC591025
HKD66	Hokkaido	2019.7	<7	Japanese beef	981	G6	LC591026	2289	P[5]	LC591027
HKD67	Hokkaido	2019.7	<7	Japanese beef	981	G6	LC591028	2289	P[5]	LC591029
HKD68	Hokkaido	2019.7	<7	Japanese beef	981	G6	LC591030	2289	P[5]	LC591031
FKS69	Fukushima	2019.8	10	Holstein	981	G6	LC591032	2289	P[5]	LC591033
FKS70	Fukushima	2019.8	10	Holstein	981	G6	LC591034	2289	P[5]	LC591035
FKS71	Fukushima	2019.8	10	Holstein	981	G6	LC591036	2289	P[5]	LC591037
FKS72	Fukushima	2019.8	8	F1 hybrid	981	G6	LC591038	2289	P[5]	LC591039
FKS73	Fukushima	2019.8	10	Japanese beef	981	G6	LC591040	2289	P[5]	LC591041
OKY75	Okayama	2019.7	13	F1 hybrid	981	G10	LC591042	2271	P[11]	LC591043
OKY76	Okayama	2019.8	10	Holstein	975	G10	LC591044	2271	P[11]	LC591045
OKY77	Okayama	2019.8	13	Holstein	975	G10	LC591046	2271	P[11]	LC591047
OKY78	Okayama	2019.9	9	Holstein	981	G10	LC591048	2271	P[11]	LC591049
HKD79	Hokkaido	2019.10	5	Holstein	981	G6	LC591050	2289	P[5]	LC591051
HKD80	Hokkaido	2019.10	5	Holstein	981	G6	LC591052	2289	P[5]	LC591053
HKD81	Hokkaido	2019.10	5	Holstein	981	G6	LC591054	2289	P[5]	LC591055
HKD82	Hokkaido	2019.10	5	Holstein	981	G6	LC591056	2289	P[5]	LC591057
HKD83	Hokkaido	2019.11	13	Japanese beef	981	G6	LC591058	2283	P[5]	LC591059
HKD84	Hokkaido	2020.1	7	Japanese beef	981	G6	LC591060	2280	P[5]	LC591061
HKD85	Hokkaido	2019.12	5	Japanese beef	981	G6	LC591062	2247	P[5]	LC591063
HKD86	Hokkaido	2020.1	7	Japanese beef	981	G6	LC591064	2289	P[5]	LC591065
HKD87	Hokkaido	2020.2	12	Japanese beef	981	G6	LC591066	2289	P[5]	LC591067
HKD88	Hokkaido	2020.2	13	Holstein	981	G10	LC591068	2268	P[11]	LC591069
HKD89	Hokkaido	2020.2	4	Holstein	981	G6	LC591070	2289	P[5]	LC591071
HKD91	Hokkaido	2020.5	14	F1 hybrid	981	G6	LC591072	2292	P[5]	LC591073
FKS92	Fukushima	2020.2	9	F1 hybrid	957	G6	LC591074	2292	P[5]	LC591075
FKS93	Fukushima	2020.2	12	Holstein	N.D.	Gx	-	2235	P[11]	LC591076
FKS94	Fukushima	2020.3	10	F1 hybrid	981	G10	LC591077	N.D.	P[x]	-
FKS95	Fukushima	2020.3	8	F1 hybrid	954	G6	LC591078	2136	P[5]	LC591079
MYZ96	Miyazaki	2020.2	8	Japanese beef	981	G6	LC591080	2220	P[5]	LC591081
MYZ97	Miyazaki	2020.2	10	Japanese beef	981	G6	LC591082	2292	P[5]	LC591083
KGS98	Kagoshima	2020.3	5	Japanese beef	981	G6	LC591084	2280	P[5]	LC591085
OKY100	Okayama	2019.12	7	Japanese beef	981	G6	LC591086	2286	P[5]	LC591087
OKY101	Okayama	2020.2	9	Holstein	N.D.	Gx	-	2200	P[11]	LC591088
OKY102	Okayama	2020.4	21	Japanese beef	981	G6	LC591089	2154	P[5]	LC591090
OKY103	Okayama	2020.4	6	Japanese beef	981	G6	LC591091	2292	P[5]	LC591092
OKY104	Okayama	2020.4	5	Japanese beef	981	G6	LC591093	2286	P[5]	LC591094
OKY105	Okayama	2020.4	14	Holstein	981	G6	LC591095	N.D.	P[x]	-
OKY106	Okayama	2020.4	5	Japanese beef	981	G6	LC591096	2292	P[5]	LC591097
OKY107	Okayama	2020.4	6	Japanese beef	981	G6	LC591098	2286	P[5]	LC591099
OKY108	Okayama	2020.5	10	Japanese beef	981	G6	LC591100	2286	P[5]	LC591101
OKY109	Okayama	2020.5	6	Holstein	921	G6	LC591102	N.D.	P[x]	-

^a^ G6, G10, and Gx are shown in light-blue, green, and blue, respectively; b P[5], P[11], and P[x] are shown in orange, pink, and red, respectively.

**Table 2:** Summary of neutralization antibody titers of two anti-bovine rotaviruses A (RVA) immunoglobulins Ys (IgYs) and one control IgY against ten bovine RVA isolates.

IgY	Genotype	Neutralization Antibody titer of Three IgYs against Bovine RVA with Different Genotypes ^a^									
		SMN-1	HKD18	SMN35	HKD6	HKD7	HKD17	KK-3	OKY31	MYG-1	Dai-10
		G6P[1]	G6P[5]	G6P[5]	G6P[11]	G6P[11]	G6P[11]	G10P[11]	G10P[11]	G8P[14]	G24P[33]
Anti-SMN35 IgY	G6P[5]	2560	2560	5120	1280	1280	1280	160	80	80	<80
Anti-OKY31 IgY	G10P[11]	<160	320	160	640	640	640	2560	2560	<80	<80
Control IgY		<20	<20	<20	<20	<20	<20	<20	<20	<20	<20

^a^ IgY titer is expressed as dilution factor of 1 g hyperimmunized IgY powder that showed inhibition of cytopathic effects (CPE) or, in other words, that protected >50% of cells from CPE. Results are presented as the mean of three independent experiments.

## Supplementary Materials

The following are available online at https://www.mdpi.com/1999-4915/12/12/1386/s1, Table S1: Ten bovine rotavirus A strains used in this study for cross-reactivity of anti- bovine rotavirus A immunoglobulin Y in neutralization assay.
