# Supplementary material for: Development of Genotype-Specific Anti-Bovine Rotavirus A Immunoglobulin Yolk Based on a Current Molecular Epidemiological Analysis of Bovine Rotaviruses A Collected in Japan during 2017–2020
Source: Viruses. 2020 Dec 3;12(12):1386. doi: 10.3390/v12121386 (PMC7761885; doi:10.3390/v12121386)
Supplement: Supplementary file 1 [file viruses-12-01386-s001.pdf]

Supplementary Table 1. Ten bovine rotavirus A strains used in this study for cross-reactivity of anti- bovine rotavirus A immunoglobulin Y in neutralization assay.

Strains [a]	Origin	Age [Day]	Area	Collection year	G and P genotype
SMN-1	Japanese beef	<10	Shimane	1977	G6P[1]
HKD6	Holstein	7	Hokkaido	2017	G6P[11]
HKD7	Holstein	9	Hokkaido	2017	G6P[11]
HKD17	Holstein	12	Hokkaido	2017	G6P[11]
HKD18	Holstein	< 7	Hokkaido	2018	G6P[5]
SMN35	Japanese beef	13	Shimane	2018	G6P[5]
KK-3	Japanese beef	Unknown	Unknown	1983	G10P[11]
OKY31	F1 hybrid	9	Okayama	2017	G10P[11]
Dai-10	Asymptomatic calf	Unknown	Hyogo	2007	G24P[33]
MYG-1	Adult cow	Unknown	Miyagi	2017	G8P[14]

[a] HKD6, HKD7, HKD17, HKD18, OKY31, and SMN35 were isolated in this study. KK-3 and SMN-1, originally providing from the National Institute of Animal Health (Tsukuba, Ibaraki, Japan), were maintained in our laboratory. The two remaining bovine RVA strains (MYG-1 and Dai-10) were kindly gifted from Dr. Matsuo, and Dr. Sugiyama from Sendai Livestock Hygiene Center (Sendai, Miyagi, Japan), and Gifu University (Gifu, Gifu, Japan), respectively.
